# Retraction: Correction: Chitosan/xanthan gum based hydrogels as potential carrier for an antiviral drug: fabrication, characterization, and safety evaluation

**DOI:** 10.3389/fchem.2026.1945290

**Published:** 2026-07-28

**Authors:** Frontiers Editorial Office

The journal retracts the 4 January 2023 article cited above.

This Correction article is retracted because the associated original article (https://doi.org/10.3389/fchem.2020.00050) has been retracted. The Correction is therefore void.

